# Emergency Ultrasound Predicting the Need for Therapeutic Laparotomy among Blunt Abdominal Trauma Patients in a Sub-Saharan African Hospital

**DOI:** 10.1155/2014/793437

**Published:** 2014-02-13

**Authors:** P. C. M. Musiitwa, M. Galukande, S. Bugeza, H. Wanzira, R. Wangoda

**Affiliations:** ^1^Department of Surgery, College of Health Sciences, Makerere University, Kampala, Uganda; ^2^Department of Radiology, College of Health Sciences, Makerere University, Kampala, Uganda; ^3^Infectious Diseases Research Collaboration, Makerere University, Kampala, Uganda

## Abstract

*Background*. The trauma burden globally accounts for high levels of mortality and morbidity. Blunt abdominal trauma (BAT) contributes significantly to this burden. Patient's evaluation for BAT remains a diagnostic challenge for emergency physicians. SSORTT gives a score that can predict the need for laparotomy. The objective of this study was to assess the accuracy of SSORTT score in predicting the need for a therapeutic laparotomy after BAT. *Method*. A prospective observational study. Eligible patients were evaluated for shock and the presence of haemoperitoneum using a portable ultrasound machine. Further evaluation of patients following the standard of care (SOC) protocol was done. The accuracy of SSORTT score in predicting therapeutic laparotomy was compared to SOC. *Results*. In total, 195 patients were evaluated; M : F ratio was 6 : 1. The commonest injuries were to the head 80 (42%) and the abdomen 54 (28%). A SSORTT score of >2 appropriately identified patients that needed a therapeutic laparotomy (with sensitivity 90%, specificity 90%, PPV 53%, and NPV 98%). The overall mortality rate was 17%. *Conclusion*. Patients with a SSORTT score of 2 and above had a high likelihood of requiring a therapeutic laparotomy. SSORTT scoring should be adopted for routine practice in low technology settings.

## 1. Introduction

Trauma burden globally is on the increase accounting for high levels of mortality and morbidity. It is the second largest single cause of disease accounting for 16%, only second to parasitic and infectious diseases at 23%. WHO also estimates that, by the year 2020, trauma will be the first or second leading cause of “years of productive life lost” for the entire world population in both developed and developing world [1].

Blunt abdominal trauma (BAT) is injury to intra-abdominal or retroperitoneal viscera as a result of a non-penetrating force. It is commonly due to rapid deceleration or acceleration in combination with shearing and rotational forces [2, 3]. Common manifestations are hemorrhage and visceral perforation, either intraperitoneal or retroperitoneal; morbidity and mortality are associated with delay in diagnosis and treatment of BAT [4–6].

Common causes include road traffic crushes (RTC), industrial accidents, falls from heights, sporting, and farm accidents (in rural areas) and sometimes the cause of BAT is unknown, for example, with child abuse and domestic violence [7, 8].

Evaluation of patients with BAT is often a diagnostic challenge for emergency physicians and trauma surgeons [9, 10]. Uncontrolled hemorrhage is responsible for over 50% of trauma related deaths [11–13].

Other times overly aggressive management can lead to nontherapeutic laparotomy. The negative laparotomy rate is 16% and negative laparotomy carries the risk of incisional hernia and small bowel obstruction over time. Emergent sonography in form of Focused Abdominal Sonography for Trauma (FAST) has emerged and been embraced as a rapid, noninvasive, and accurate method of evaluating blunt abdominal trauma that can be easily used by emergency room clinicians and trauma surgeons [14–17]. FAST is a rapid, four-view ultrasound examination carried out during the primary survey that assesses for haemoperitoneum, haemothorax, and haemopericardium.

A newer dimension of emergency sonography is the Sonographic Scoring for Operating Room Triage in Trauma (SSORTT score) which was developed in a trauma center in the United States of America with the aim of enabling emergency room clinicians to quickly and reliably predict which patients require an emergent laparotomy. It has three components: (1) FAST findings, (2) systolic blood pressure and, (3) pulse rate which together give a score that can predict whether patient would most likely not require a therapeutic laparotomy.

The objective of this study therefore was to determine whether specific SSORTT scores are good predictors of a need for a therapeutic laparotomy among blunt abdominal trauma patients in a low resource settings.

## 2. Methods

### 2.1. Study Design

A prospective observational cohort study carried out from December 2012 to April 2013.

### 2.2. Study Setting

The study was conducted in the 24/7 A and E department of Mulago National referral hospital situated in Kampala, Uganda. This is a fully fledged unit with a medical and surgical emergency wing, two operating rooms, an X-ray facility, ultrasound facility, resuscitation room with 3 beds, and a 26-bed holding emergency ward; adjacent to it are the blood bank, hematology, microbiology, and chemistry laboratories.

It receives about 1500 trauma patients per month, 300 with suspected torso injuries and 20 with actual torso injuries [18].

### 2.3. Study Procedure

Upon arrival in the A&E unit patients with suspected BAT were screened.

BAT was defined as injury to intra-abdominal viscera or retroperitoneal as a result of a nonpenetrating object or force indicated by presence of torso abrasions and bruises, abdominal tenderness among multiply injured patients with long bone fractures, fracture pelvis, and spine injuries.

Patients meeting these eligibility criteria were recruited and subsequently informed written consent was obtained. All patients were adequately resuscitated and investigated as per the standard of care protocol. Patients with subcutaneous emphysema over the abdomen in whom abdominal assessment with sonography would be difficult to interpret were excluded.

Assessment was done using ATLS protocol. History and physical examination findings were recorded on pre-coded questionnaires. Patients suspected to be having BAT were positioned in supine position and underwent FAST using SonoSite TITAN portable ultrasound machine with a transducer frequency ranging from 3.5 to 5 MHz. A cumulative sum of the three parameters ultrasound score, systolic blood pressure, and pulse rate were determined and used as the SSORTT score.

Patient then underwent secondary survey, which was followed by other routine investigations and management according to the hospital guidelines. As and when appropriate patients were sent to the emergency radiology room for a full ultrasound scan and abdominal X-ray. All admitted patients were followed up for three days to determine whether they had laparotomy or not.

### 2.4. Study Variables

These included the SSORTT score, age, sex, and mechanism of injury, for example, RTC. In addition and therapeutic laparotomy, nontherapeutic laparotomy, and outcomes of nonoperative management (lived/died).

### 2.5. Data Collection, Management, and Analysis

Data were collected using pretested questionnaires and double entered, coded, and cleaned using Epi data version 5.3.2 software. Stored data were exported to Stata version 12 for analysis. Accuracy of the SSORTT score in predicting patients that would need therapeutic laparotomy was determined by calculating sensitivity, specificity, PPV, NPV, and ROC curve of SSORTT score. ROC curve was used to determine diagnostic accuracy of SSORTT score for predicting the need for therapeutic laparotomy.

### 2.6. Quality Control

The investigator(s) underwent didactic tutorials on FAST also performed at least 30 ultrasound scans to determine presence of haemoperitoneum prior to starting the study.

The ultrasound machine was maintained and calibrated by hospital staff from the department of radiology. We stored the examinations images (sonographs) and had them reread (validated) by a consultant radiologist.

### 2.7. Ethical Considerations

Written informed consent for patients above 18 years and assent for those less than 18 years were obtained from the participants. Ethical approval was obtained from the Research and Ethics Committee of the College of Health Sciences of Makerere University.

## 3. Results

**Figure 1:**
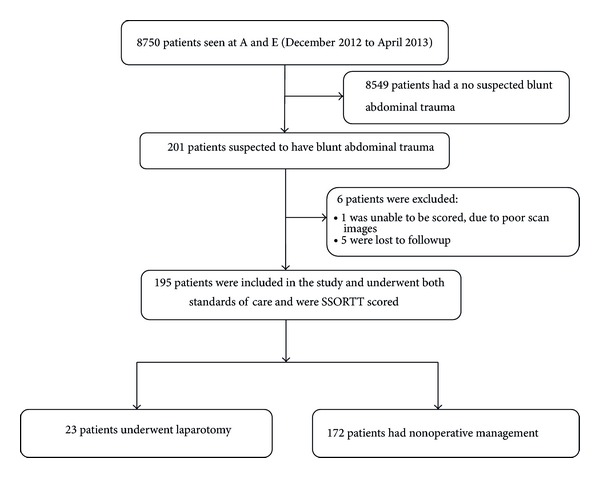
Study profile.

A total of 195 trauma patients clinically suspected to have sustained BAT were subjected to emergency ultrasound scanning and were SSORTT scored at the A&E unit of Mulago Hospital from December 2012 to April 2013. The study profile is shown in Figure 1. The patients' characteristics and laparotomy findings are outlined in Table 1.

The male : female ratio was 6 : 1. Age group between 20 and 40 years comprised majority of patients 153 (79%). Road traffic crashes (RTC) were the leading cause of injury in this study accounting for 59%.Others causes included assault 32%, falls 7%, and others 2% as shown in Table 1.

Most of patients with BAT had clinically suffered injuries to the head, abdomen, or limbs.

Table 1 shows the distribution of SSORTT scores by intervention. 20/23 (86%) patients who had therapeutic laparotomy had a SSORTT score of  ≥3. 156/173 (90%); patients who had nonoperative management had a SSORTT score of ≤2.

Table 2 shows the laparotomy predictions at selected cut offs.

Table 3 shows that the majority of patients who had a laparotomy had high Glasgow coma scores. A small proportion had Glasgow coma score < 13.

More than 80% of the patients with abdominal tell-tale signs and head injury were subjected to a laparotomy.

Majority of the patients with head injury were subjected to nonoperative management.

Among therapeutic laparotomy patients, massive haemoperitoneum was the commonest finding (47%), followed by solid organ injury at 35% and gut perforation at 6%.

Among nontherapeutic laparotomy retroperitoneal hematoma and <grade3 solid organs injury were findings.

The general trend for sensitivity at the various cut-off levels of the SSORTT score was that it reduced with an increasing score; see Table 3.

Values of specificity gradually increase as

 at SSORTT score 0, sensitivity = 91% and specificity = 82%;
 at SSORTT score 6, sensitivity = 0% and specificity = 100%;
 at SSORTT score 0, PPV = 39% and NPV = 99%;
 at SSORTT score 6, PPV = 0% and NPV = 89%.

### 3.1. Accuracy 

For Accuracy of the Test, the following ranking was considered:

 .90–1 = excellent (A);
 .80–.90 = good (B);
 .70–.80 = fair (C);
 .60–.70 = poor (D);
 .50–.60 = fail (F).

**Figure 2:**
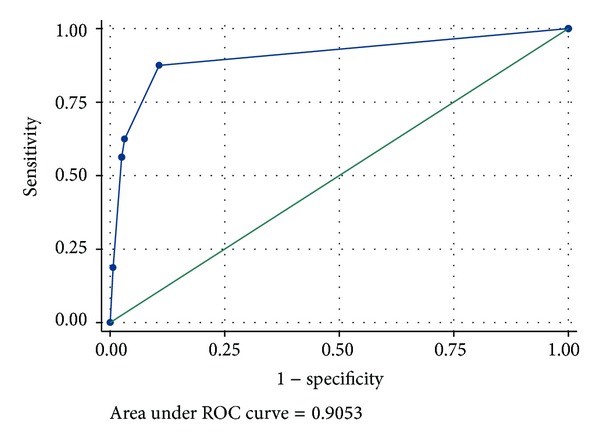
ROC curve for SSORTT score.

The area under the ROC curve measured accuracy; see Figure 2.

The closer the curve follows the left-hand border and then the top borders of the ROC space, the more accurate the test is. The area under ROC curve measured 0.91 a high value for accuracy.

The presence of signs which included bruises, abrasions, lacerations, and cuts did not predict staying alive or dying. The patients who had a laparotomy were less likely to die OR 1.64 though it was not statistically significant *P *= 0.370.

Of 23 patients that had laparotomy 21 patients had therapeutic laparotomy and 2 being nontherapeutic, one had a moderately large retroperitoneal haematoma and the other had a grade two splenic laceration with a haemoperitoneum of about 500 cc. Of the 172 patients that did not undergo laparotomy 28 patients passed away and the rest were either discharged or still admitted by day three. Of the 28 patients, 18 patients were multiply injured with severe extra-abdominal injuries. This can be shown in Table 3 where majority of patient; that is; 18 patients that died; had SSORTT 0 and 23 patients had Glasgow coma score less than 8 indicating presence of severe extra-abdominal injuries.

16 patients did not have a postmortem done because the relatives declined. Haemoperitoneum was found in 3 patients and 7 patients had blunt force trauma with extra-abdominal injury.

Majority of patients that died had a SSORTT score of 0 (55%).

Majority of deaths were in the severely deranged GCS range, that is, 3–8.

Seven patients with intra-abdominal fluid died.

All patients that passed away with intra-abdominal fluid had scores of >2.

## 4. Discussion

We set out to determine the diagnostic accuracy of emergency ultrasound scanning using the SSORTT score in predicting the need for a therapeutic laparotomy for blunt abdominal trauma patients. The SSORTT score was defined as a sum of the ultrasound haemoperitoneum score, systolic blood pressure, and pulse rate [19, 20]. Patients with a SSORTT score of 2 and above had a high likelihood of requiring a therapeutic laparotomy and those below that were unlikely to need one.

SSORTT scoring had excellent diagnostic accuracy for identifying patients that needed or did not need a therapeutic laparotomy with sensitivity of 91% and specificity of 90%.

The findings of this study are comparable to findings by Manka et al. where patients with score of less than 1 were less likely to require a laparotomy [21].

The youths were mostly affected because they fall in the most active age bracket exposed to most injury risks [8, 22, 23].

Motorcycle accidents accounted for most (62%) of the injuries; the use of motorcycles a cheap means of transportation is on the increase in many countries in Africa and are a leading cause of blunt abdominal trauma [22–28].

Many studies have demonstrated that physical examinations are unreliable in assessing trauma patient especially those with neurological injury (brain or spinal cord), and in those with painful distracting injuries such as long bone or pelvic fractures, or in those with alcohol or other intoxicants in their systems [29, 30].

Eighty patients suffered head injuries (42%) and 50 (26%) had potentially distracting limb extremity injuries. In the absence of diagnostic aids a proportion of the severely injured would be vulnerable to delays in diagnosis of major intra-abdominal visceral injuries. We contend that proper use of ultrasound in the emergency room in early identification of life threatening injuries like intra-abdominal hemorrhage, haemothorax, or haemoperitoneum may save lives in low technology settings. The additional advantage of sonographers is that it is noninvasive compared to techniques like peritoneal lavage which we many times use in the absence of sonography [22].

We found that 23 patients (12%) received laparotomy and of these 21 were therapeutic, that is, surgically correctable lesions. Massive haemoperitoneum with solid organ injury was the commonest finding at laparotomy with ruptured spleen being the commonest solid organ injured followed by liver lacerations. There were two cases of gut perforations, one urinary bladder rupture and one with mesenteric tears.

This pattern of injuries is consistent with many other studies done in the country and the region [6, 22–26, 31, 32].

Five patients of the 23 that had laparotomy passed away, due to anesthesia related complications and aneamia or coagulopathy due to shortage of blood. The laparotomy rate in this study was 11% and the overall mortality rate was 17%, comparable to previous studies [22, 23] which reported a mortality rate between 10 and 30%.

### 4.1. Study Limitations

This study was not without limitations; the sensitivity and specificity of emergency US scanning could have been affected by possible sonography operator differences; however some studies show that FAST can be done reliably by both radiologist and nonradiologists [15, 33, 34].

In the calculation of the SSORTT score, ideally blood pressure and pulse rate should be obtained at the beginning of resuscitation. However, often blood pressure and pulse rates would be taken after fluid infusions that were started to resuscitate patients; this could be assumed to influence the SSORTT score leading to an under estimation of severity of injury. Postmortem to determine cause of death were not done for various reasons like attendants declining.

## 5. Conclusion 

SSORTT score is a noninvasive, reproducible test that can be performed easily and reliably predicts the need for a therapeutic laparotomy among blunt abdominal trauma patients. It should be adopted for routine use in low technology settings.

## Figures and Tables

**Table 1:** Distribution of baseline characteristics and laparotomy findings for the 195 participants.

Characteristics	Participants	
	Number	Percentage
Age groups		
<20	22	11
20–40	153	79
41–60	18	9
>60	2	1
Gender		
Male	168	86
Female	27	14
Time since injury in hours*		
<1	20	10
1-2	84	43
>2	73	37
Unknown	18	10
Mechanism of injury*		
Road traffic crash	116	59
Assault	62	32
Fall	13	6
Others	4	2
Therapeutic laparotomy		
Massive haemoperitoneum	16	47
Grade 4/5 solid organs injury	12	35
Gut perforation	2	6
Mesenteric tears	1	3
Bladder injury	1	3
Nontherapeutic laparotomy		
Retroperitoneal haematoma	1	3
Grade 3 solid organs injury	1	3

SSORTT score	Laparotomy *N* = 23	No laparotomy *N* = 172
	Number (%)	Number (%)

0	2 (9)	142 (83)
1	0	13 (8)
2	1 (4)	1 (1)
3	5 (22)*	12 (7)
4	6 (26)	3 (2)
5	3 (13)	1 (1)
6	6 (26)*	1 (0)

*Two participants had nontherapeutic laparotomy with scores of 3 and 6.

**Table 2:** Laparotomy predictions at selected cutoffs.

SSORTT score	Standard of care		Sensitivity	Specificity	PPV (%)	NPV (%)
	Laparotomy	No laparotomy				
*Score cutoffs *						
At 0						
Laparotomy	19	30				
No laparotomy	2	142	91	82	39	99
≥1						
Laparotomy	19	17				
No laparotomy	2	155	90	90	53	99
≥2						
Laparotomy	18	16				
No laparotomy	3	156	86	91	53	98
≥3						
Laparotomy	14	4				
No laparotomy	8	168	67	98	78	96
≥4						
Laparotomy	8	1				
No laparotomy	14	171	38	99	89	93
≥5						
Laparotomy	5	0				
No laparotomy	17	172	24	100	100	91
At 6						
Laparotomy	0	0				
No laparotomy	22	172	0	100	0	87

**Table 3:** Comparison of the fatalities and survivors.

Injuries	Patient status		OR (95% CI)	*P* value
	Alive	Died		
	Number (%)	Number (%)		
Bruises	66 (47)	21 (66)	0.5 (0.2–1.0)	0.063
Abrasions	30 (21)	8 (25)	0.8 (0.3–2.0)	0.661
Lacerations	79 (56)	18 (56)	1.0 (0.5–2.2)	0.985
Cuts	10 (7)	3 (9)	0.7 (0.2–2.9)	0.667
Burns	2 (1)	1 (3)	0.5 (0.1–5.1)	0.519
Abdominal	43 (30)	7 (22)	1.6 (0.6–3.9)	0.334
Chest	16 (11)	6 (19)	0.6 (0.2–1.6)	0.262
Others	120 (86)	23 (74)	2.2 (0.9–5.6)	0.100
Standard of care laparotomy	17 (11)	6 (18)	1.6 (0.6–4.9)	0.370
SSORTT score				
0	123	18	—	—
1	10	3	—	—
2	2	0	—	—
3	11	6	—	—
4	8	1	—	—
5	3	1	—	—
6	2	4	—	—
Glasgow coma score				
Severe	13	21	—	—
Moderate	21	9	—	—
Mild	125	3	—	—

## Abbreviations

AE: Accident and emergency
BAT: Blunt abdominal trauma
FAST: Focused abdominal sonography for trauma
RTC: Road traffic crashes
SSORTT: sum of ultrasound score, systolic blood pressure, and pulse rate.
